# Extracellular ATP acts as a damage-associated molecular pattern (DAMP) signal in plants

**DOI:** 10.3389/fpls.2014.00446

**Published:** 2014-09-03

**Authors:** Kiwamu Tanaka, Jeongmin Choi, Yangrong Cao, Gary Stacey

**Affiliations:** ^1^Department of Plant Pathology, Washington State UniversityPullman, WA, USA; ^2^Division of Plant Sciences and Biochemistry, Christopher S. Bond Life Sciences Center, University of MissouriColumbia, MO, USA

**Keywords:** extracellular ATP, DAMPs, immune defense, wound healing, symbiosis and immunity

## Abstract

As sessile organisms, plants have evolved effective mechanisms to protect themselves from environmental stresses. Damaged (i.e., wounded) plants recognize a variety of endogenous molecules as danger signals, referred to as damage-associated molecular patterns (DAMPs). ATP is among the molecules that are released by cell damage, and recent evidence suggests that ATP can serve as a DAMP. Although little studied in plants, extracellular ATP is well known for its signaling roles in animals, including acting as a DAMP during the inflammatory response and wound healing. If ATP acts outside the cell, then it is reasonable to expect that it is recognized by a plasma membrane-localized receptor. Recently, DORN1, a lectin receptor kinase, was shown to recognize extracellular ATP in *Arabidopsis*. DORN1 is the founding member of a new purinoceptor subfamily, P2K (P2 receptor kinase), which is plant-specific. P2K1 (DORN1) is required for ATP-induced cellular responses (e.g., cytosolic Ca^2+^ elevation, MAPK phosphorylation, and gene expression). Genetic analysis of loss-of-function mutants and overexpression lines showed that P2K1 participates in the plant wound response, consistent with the role of ATP as a DAMP. In this review, we summarize past research on the roles and mechanisms of extracellular ATP signaling in plants, and discuss the direction of future research on extracellular ATP as a DAMP signal.

## ENDOGENOUS DANGER SIGNALS, DAMPs IN PLANTS

Multicellular organisms have assembled complex signaling networks that mediate specific and dynamic responses following various environmental stimuli. Among these are mechanisms that recognize a potentially life-threatening event as a danger signal. Danger signals include exogenous, enemy-derived signal molecules, e.g., pathogen-associated molecular patterns (PAMPs), which are recognized by pattern recognition receptors to activate immune responses. In addition, endogenous molecules and fragments from damaged cells and tissues can also be recognized as danger signals, referred to as damage-associated molecular patterns (DAMPs). Multicellular organisms use DAMPs for damage-self recognition to evoke immune inflammatory responses and damage healing independent of but in cooperation with exogenous danger signals (Matzinger, 2007).

Because of their sessile nature, plants are continuously exposed to various stresses caused by changes in the environment and attacks by other organisms, i.e., abiotic and biotic stresses. Therefore, plants require sophisticated surveillance systems to detect a variety of danger signals. Indeed, plants have evolved a large number of receptor kinases (e.g., >600 genes in *Arabidopsis*), most of which are likely involved in the response to different stresses, based on the observation that many duplication events have occurred in the genes involved in defense responses and disease resistance (Shiu et al., 2004). These plant receptors recognize not only exogenous danger signals such as PAMPs and herbivore-associated molecule patterns (HAMPs) but also endogenous danger signals such as DAMPs. A number of PAMPs have been identified, and their recognition systems and downstream signaling events are well studied and understood (Zipfel, 2014). In contrast, only a few DAMPs have been extensively studied (**Table 1**) and the details regarding their recognition and signaling mechanisms remain unclear. To date, few receptors have been identified that specifically recognize DAMP signals (**Table 1**). Further studies are needed to clarify each of their signaling mechanisms.

**Table 1:** Damage-associated molecular patterns (DAMPs) in plants.

Type	Molecule/fragment	Representative reference	Receptor
Nucleotides	ATP	Jeter et al. (2004)	P2K1/DORN1 Choi et al. (2014)
	NAD(P)H	Zhang and Mou (2009)	n.d.
	DNA	Wen et al. (2009)	n.d.
Saccharides	Sucrose	Heil et al. (2012)	n.d.
	Oligogalacturonic acid (OGA)	Reymond et al. (1995)	WAK1 Decreux et al. (2006), Brutus et al. (2010)
Volatile organic compounds	Green leaf volatiles	Paré and Tumlinson (1999)	n.d.
Peptides	Systemin*	Ryan and Pearce (2003)	SR160 Scheer and Ryan (2002)
	Pep914/890**	Yamaguchi et al. (2011)	n.d.
	Subtilase peptide (SubPep)**	Pearce et al. (2010)	n.d.
	Hydroxyproline-rich glycopeptides	Pearce et al. (2007)	n.d.
	Elicitor peptides (Peps)	Huffaker et al. (2006)	PEPR1/2 Yamaguchi et al. (2006, 2010)

(*) Solanaceae specific peptide; (**) legume specific peptide; (n.d.) not determined.

**FIGURE 1:**
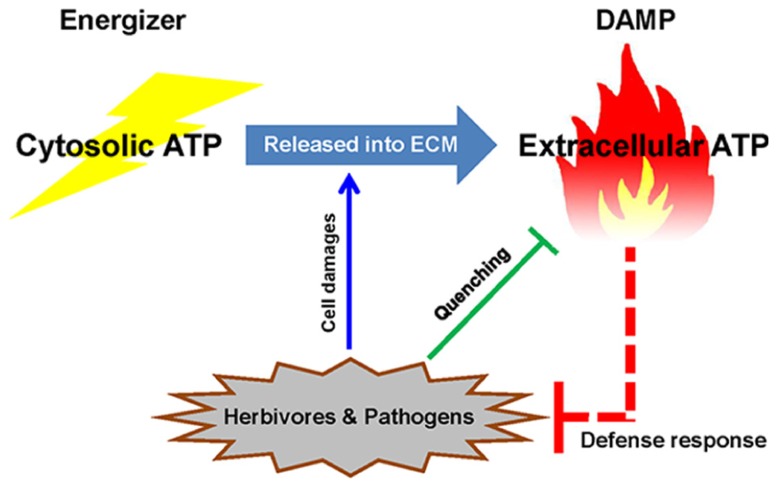
**Extracellular ATP as a danger signal in plants.** Once energizer ATP is released from the cytosol to the extracellular milieu (ECM) by cellular damage (e.g., herbivore or pathogen attack), ATP acts as a DAMP signal to trigger plant defense responses. The plant pathogens and pests have strategies to interfere with extracellular ATP signaling by hydrolyzing the molecule, suggesting that ATP plays an important role in the plant defense response.

Extracellular ATP is one of the well studied DAMP signals in both animals and plants. Although ATP is well recognized as a source of high energy phosphate bonds to support cellular metabolism, once ATP is released from cells following cellular damage, it acts as a DAMP signal (**Figure 1**). ATP is a good choice for such a role since cells contain a high concentration of ATP (1–10 mM), which is highly reactive and involved in more chemical reactions than any other compound except H_2_O. In animals, extracellular ATP has been studied for over 60 years. The released ATP is recognized by plasma membrane-localized purinergic receptors (P2X and P2Y) that involve a wide range of animal physiology (Khakh and Burnstock, 2009). In plants, a number of earlier studies reported that extracellular ATP plays essential roles in plant growth and development (Tanaka et al., 2010a). The recent discovery of the plant P2K1 (DORN1) receptor by Choi et al. (2014) demonstrated that extracellular ATP also serves as a DAMP signal in plants. For example, most ATP-responsive genes were also regulated in response to a wound treatment. As would be expected, *dorn1* mutant plants showed a reduced transcriptional response to both ATP treatment and wounding. Similarly, overexpression of *DORN1* resulted in an elevated response to both ATP and wounding. These data strongly suggest that ATP plays an important role in response to wounding, which is mediated by ATP recognition by the P2K1 (DORN1) receptor.

Detailed analysis of ATP-induced genes by Cao et al. (2014) suggested that extracellular ATP signaling, mediated by P2K1, was also involved in plant responses to a variety of stresses. The suppression of ATP-hydrolyzing enzymes, apyrases (AtAPY1 and AtAPY2), which results in an elevation of extracellular ATP levels, induced the expression of genes involved in stress responses (Lim et al., 2014). Therefore, extracellular ATP likely plays a role as a central danger signaling during a variety of plant stress responses.

## ATP IS RELEASED INTO THE EXTRACELLULAR MILIEU AS A DAMP SIGNAL

**FIGURE 2:**
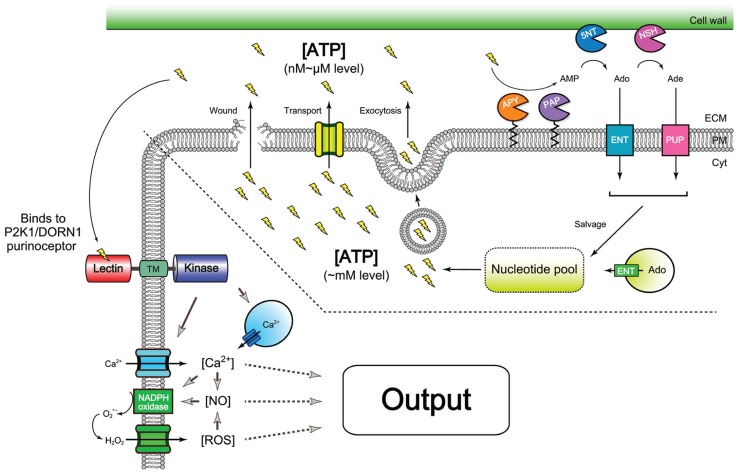
**Overview of release, homeostatic control, and signaling of extracellular ATP.** Wounding (i.e., cell breakage) is a good example of the release of ATP molecules shown as “lightning” symbols. In addition, a variety of stimuli, e.g., abiotic and biotic stresses can induce ATP release (see **Table 1** in detail). These events are suggested to be mediated by an ABC transporter (Thomas et al., 2000), PM-ANT1 transporter (Rieder and Neuhaus, 2011), or exocytotic secretion (Kim et al., 2006). Once cellular ATP (1–10 mM) appears in the apoplast, ATP becomes a signal, i.e., a DAMP signal. The released ATP is then recognized by plasma membrane-localized purinergic receptor, P2K1 (DORN1; left side), which in turn can elevate cytosolic free Ca^2+^ concentrations, [Ca^2+^] (Choi et al., 2014). This causes production of nitric oxide (NO) or ROS (Song et al., 2006; Demidchik et al., 2009). The second messenger trio, Ca^2+^, NO, and ROS activates cellular signaling pathways that eventually lead to induction of various physiological responses, including activation of gene expression. The concentration of extracellular ATP is modulated through the action of ectoapyrases (APY; Lim et al., 2014) and other phosphatases, e.g., purple acid phosphatases (PAP; Liang et al., 2010). After hydrolyses, the end product AMP is further processed to adenosine (Ado) by hypothetical 5′-nucleotidases, 5NT (Riewe et al., 2008) and further to adenine (Ade) by nucleoside hydrolases, NSH, e.g., NSH3 (Jung et al., 2011). Both adenosine and adenine can be reimported into cells by equilibrative nucleoside transporters, ENT, e.g., ENT3 (Bernard et al., 2011) and hypothetical purine permeases (PUP), respectively, and then enter the salvage pathway (Riewe et al., 2008). In other respects, vacuole nucleosides from RNA degradation are exported into cytosolic area by another ENT, e.g., ENT1 (Bernard et al., 2011), that also contributes to cytosolic nucleotide pools. The figure was reconstructed from several schemes in the previous publications (Riewe et al., 2008; Demidchik et al., 2009; Tanaka et al., 2010a; Cao et al., 2014; Möhlmann et al., 2014). Abbreviations: Cyt, cytoplasm; PM, plasma membrane; ECM, extracellular milieu.

The presence of extracellular ATP in plants was detected under various conditions (**Table 2**), which suggests the existence of mechanisms by which this energy molecule is released into the apoplast. Several mechanisms have been proposed for mediating ATP release (**Figure 2**). For example, similar to the mechanism of ATP release at animal neuronal synapses, ATP appears to be released at sites of active growth via vesicular exocytosis (Kim et al., 2006). AtPGP1, an ABC transporter, and PM-ANT1, a plasma membrane-localized nucleotide transporter were shown to export intracellular ATP into the extracellular milieu (ECM; Thomas et al., 2000; Rieder and Neuhaus, 2011). However, it remains unclear how these ATP-releasing systems are controlled by environmental stimuli.

**Table 2:** Release of extracellular ATP during plant growth, development, and stress responses.

Category	Type of stress	Stimulus	Organism	Tissue	ATP concentration or fold change (peak time)	Reference
Abiotic stress	Mechanical stimulation	Shaking at 150 rpm	*Arabidopsis*	Whole seedlings	3-fold (2 min)	Jeter et al. (2004)
		Touching by a pipette tip	*Arabidopsis*	Root and shoot tips	10–15 nM (1 min)	Weerasinghe et al. (2009)
		Wounding	*Arabidopsis*	Seedling roots	70–80 nM (15 min)	Dark et al. (2011)
				Rosette leaves	25–45 μM (immediately)	Song et al. (2006)
	Osmotic stress	Sorbitol (300 mM)	*Arabidopsis*	Whole seedlings	40–50 nM (15 min)	Dark et al. (2011)
		MgCl_2_, Mg(NO_3_)_2_ (100 mM)	*Arabidopsis*	Whole seedlings	∼1.5-fold (1 h)	Kim et al. (2006)
		NaCl (100–300 mM)	*Populus euphratica*	Suspension culture	∼15 nM (5 min)	Sun et al. (2012)
			*Arabidopsis*	Whole seedlings	2.5-fold (1 min)	Jeter et al. (2004)
					60–70 nM (15 min)	Dark et al. (2011)
					∼1.5-fold (1 h)	Kim et al. (2009)
	Others	L-Glutamate (10 μM)	*Arabidopsis*	Seedling roots	25–30 nM (5 min)	Dark et al. (2011)
		Light treatment of dark-adapted leaves	*Arabidopsis*	Guard cells	3.5-fold (10 min)**	Clark et al. (2011)
		Abscisic acid (10 μM)	*Arabidopsis*	Seedling roots	20–30 nM (10 min)	Dark et al. (2011)
				Guard cells	4.5-fold (5 min)**	Clark et al. (2011)
Biotic stress	Pathogen elicitors	Chitin mixture (100 μg/mL)	*Medicago truncatula*	Root hair	- (30 min)*	Kim et al. (2006)
		Yeast extract (100 μg/mL)	*Salvia miltiorrhiza*	Hairy root culture	70-fold (10 h)	Wu et al. (2008)
		Mycotoxin beauvericin (40 μM)	Wheat	Leaf	∼1.75-fold	Šrobárová et al. (2009)
Spontaneous	Cell growth	Cell propagation	*Arabidopsis*	Suspension culture	^-^ ***	Chivasa et al. (2005)
	Tip growth	Fiber elongation	Cotton	Ovule fiber	330 nM	Clark et al. (2010a)
		Root hair elongation	*Medicago truncatula*	Root hair	^-^ *	Kim et al. (2006)
		Pollen germination and elongation	*Arabidopsis*	Germinating pollen	∼6-fold	Wu et al. (2007)
					42.1 pmol/10^6^ pollen	Bernard et al. (2011)
					∼4.5 pmol/mg protein	Rieder and Neuhaus (2011)

(-) Indicates that ATP release was qualitatively visualized rather than quantified. ATP measurement was performed by a conventional luciferase method unless otherwise noted by asterisks as follow: (*) luciferase fused with cellulose binding domain (Kim et al., 2006); (**) luciferase fused with secretory signal peptides (Clark et al., 2011); or (***) thin layer chromatography (Chivasa et al., 2005).

Cell breakage is a simple mechanism for ATP release that could be caused by herbivore attack (i.e., wounding) or pathogen-induced cell lysis (i.e., necrosis). This released ATP would be expected to act as a DAMP. Interestingly, ∼60% of the genes induced by ATP treatment are also induced by wounding, 90% of which responded early to wounding (Choi et al., 2014). In previous studies, the ATP concentrations measured at the wound sites of *Arabidopsis* leaves (Jeter et al., 2004) and damaged roots (Dark et al., 2011) were 40 μM and 80 nM, respectively. The effective concentration for ATP signaling at damaged sites may be lower than these measurements, considering that the ATP pool is competitively shared with other ectoenzymes, such as ectophosphatases, ectokinases, and other cell wall enzymes. Given the high affinity of P2K1 for ATP recognition (Kd ∼46 nM), these levels of extracellular ATP would be sufficient to trigger P2K1-mediated ATP signaling. This finding suggests that extracellular ATP released during wounding acts as a DAMP signal and is recognized by the P2K1 receptor (**Figure 2**).

More subtle treatments of plants have also been shown to release sufficient ATP to potentially activate the P2K1 receptor. For example, nanomolar levels of ATP were released from *Arabidopsis* roots when a mild touch was applied with a force of 198 ± 178 mN (**Table 2**), which is a typical range for plants growing through soil (Weerasinghe et al., 2009). Plants can also experience mechanical stress when infected by microbes. Jayaraman et al. (2014) reported that pathogenic fungi and oomycetes exert mechanical stress by turgor pressure from penetrating pegs and hyphae. Indeed, mechanical stimulation was shown to activate disease resistance (Benikhlef et al., 2013). Interestingly, pathogen attachment causes nuclear repositioning and cytoplasmic streaming at the contact points on the plant cell (Hardham et al., 2008; Genre et al., 2009; Hardham and Blackman, 2010). These cytoskeletal arrangements are general responses caused by mechanical stimulation (Hardham et al., 2008). Considering that exogenous ATP can also cause cytoplasmic streaming in plant cells (Williamson, 1975), it is conceivable that mechanical stress following pathogen infection likely stimulates plants to release ATP as a DAMP signal, which eventually evokes cellular responses against the invaders. ATP-induced actin filament formation and cytoskeletal rearrangements are widely observed in animal cells in which ATP modulates inflammation, wound healing and angiogenesis (Kaczmarek et al., 2005). Although such a role for ATP in plants is speculative at this point, it is clear that ATP has the potential to be a missing signal that mediates many of the responses previously attributed solely to mechanical stimulation.

In addition to wounding and mechanical stress, ATP is also released, without visible cell damage, in response to treatment with biotic and abiotic stress agents (**Table 2**). Abiotic stimuli such as osmotic and salt stress, as well as abscisic acid (a stress-related hormone), and L-glutamate (known as a fast excitatory neurotransmitter in the mammalian nervous system) induce ATP release in plants (**Table 2**). Biotic stress agents such as pathogen-derived elicitors (i.e., chitin, yeast extract, and mycotoxin beauvericin) also trigger ATP release (**Table 2**).

Taken together, these findings clearly demonstrate that plants release ATP outside cells in response to various stresses, and the released ATP likely acts as a DAMP signal to trigger plant defenses and environmental stress responses (**Figure 2**). Further studies are necessary to understand how the ATP-releasing machinery (e.g., transporters and exocytotic vesicles) are regulated by an array of different stimuli.

## ROLE OF EXTRACELLULAR ATP AS A DAMP

### IMMUNE DEFENSE SYSTEM FOLLOWING WOUNDING AND CELL DAMAGE

Herbivore feeding, similar to physical wounding, would be expected to passively release high doses of ATP into the extracellular milieu. If this ATP triggers plant defense mechanisms, it would be expected that insects may have adapted to counter this defense. Indeed, some herbivorous caterpillars and whitefly larvae secrete saliva containing apyrases while feeding on plant leaves (Su et al., 2012; Wu et al., 2012). Interestingly, the exogenous application of purified caterpillar apyrase at the wound site suppressed glandular trichome production, which is involved in the defense against herbivores, and the expression of defense-related genes regulated by jasmonic acid and ethylene (Wu et al., 2012). A clear mechanism for this response would be that the salivary apyrases dampen wound-released ATP to restrict the plant defense response. Furthermore, a higher catalytic activity of apyrases was detected in globose insect galls on *Calliandra brevipes* (Detoni et al., 2012). Similar mechanisms for controlling extracellular ATP concentrations are observed in the interactions between animals and parasitic worms or blood sucking insects (Valenzuela et al., 2001; Da’dara et al., 2014).

Pathogen attacks cause cell damage that allows cytoplasmic ATP to leak into the extracellular space. The reduction of extracellular ATP levels was observed following plant infection by the bacterial pathogen *Pseudomonas syringae*. Interestingly, this reduction was not observed using a *P. syringae* strain lacking a canonical protein secretion system (Chivasa et al., 2009), suggesting that some effector proteins may contribute to ATP depletion. A recent study showed that adenylate kinases (which phosphorylates AMP using ATP as a phosphate donor) are secreted by *Xanthomonas oryzae* (Robin et al., 2014). These enzymes could play a role in quenching DAMP-utilized ATP during colonization. Adenylate cyclase were also shown to be important for the virulence of animal pathogens (Markaryan et al., 2001; Munier-Lehmann et al., 2003). Given that extracellular ATP was shown to be involved in cytolysis, cell adhesion, and growth of various microbes (Roberto Meyer-Fernandes, 2002; Meyer-Fernandes et al., 2004; Li et al., 2011), these pathogenic microbes may be merely protecting themselves from ATP, a very reactive molecule. Correspondingly, a recent report by Long et al. (2014) demonstrated that conidial germination, appressorium formation and pathogenicity of rice blast fungus *Magnaporthe oryzae* were significantly reduced by pretreatment with nucleotidase-specific inhibitors.

The above results suggest that pathogens and insects directly manipulate extracellular ATP levels to weaken or block plant immunity; thereby promoting colonization of the plant host (**Figure 1**).

Plants use hormone-mediated pathways, either salicylic acid, jasmonic acid and/or ethylene, to activate defense against pathogens or herbivores (Koornneef and Pieterse, 2008; Mur et al., 2013). The application of ATP induces the expression of genes encoding biosynthetic enzymes for jasmonic acid and ethylene (Jeter et al., 2004; Song et al., 2006). Interestingly, Heil et al. (2012) observed that ATP addition to slightly wounded tissue induced the secretion of extrafloral nectar in the lima bean. The secretion of extrafloral nectar is a jasmonic acid-dependent defense response, known to attract ants and other predatory insects (Heil et al., 2001). These results suggest that ATP released following wound damage activates signaling by stress hormones that eventually activates additional defense systems.

It is expected that more than two DAMPs would be present together in the plant apoplastic area at the wound site, where the signals would function together to activate defense response signaling. Oligogalacturonic acid (OGA), another DAMP generated by cell wall degradation (**Table 1**), is known to trigger cytosolic Ca^2+^ elevation, reactive oxygen species (ROS) production, and other downstream defense responses (Chandra and Low, 1997). These same responses are also triggered by ATP (Demidchik et al., 2009; Tanaka et al., 2010b). Co-treatment with both OGA and ATP resulted in an enhancement of cytosolic Ca^2+^ elevation (Jeter et al., 2004). Given that similar defense signaling and gene expression are triggered by both ATP and OGA (Rojo et al., 1999; Jeter et al., 2004), it is likely that these two signals may act synergistically to insure a robust response to wounding.

### HEALING AFTER WOUNDING AND CELL DAMAGE

Wound healing is an essential biological process composed of complex, sequential steps to restore function of damaged cells and surrounding tissues. In animals, this process starts with conserved cellular damage signals (e.g., Ca^2+^, ROS, and ATP) that diffuse into ambient tissue, surround the wounded area, and initiate immediate cellular events including cell shape changes, actomyosin formation, and immune cell recruitment (Cordeiro and Jacinto, 2013).

Plants lack cellular mobility because of the cell wall and must restore tissue through cellular regeneration at the damaged site. A recent report by Lim et al. (2014) using RNAi silencing of ectoapyrases suggested that accumulated levels of extracellular ATP caused significant changes in the expression of genes regulating wall composition and extensibility. The silenced lines showed changes in wall lignification and decreased methyl ester bonds. These results suggest that extracellular ATP induced by wounding could play an important role in wall reorganization during tissue healing.

The molecular mechanisms of the early stage, plant healing process remain largely unknown, in contrast to those of later-stage wound healing, which has been well studied (Asahina et al., 2002, 2011; Xu et al., 2006; Iwase et al., 2011). Asahina et al. (2011) noted that auxin plays an important role during communication between the repair site and other parts of the plant. Auxin accumulates on the acropetal side of the wound while lower levels are found on the basipetal side. Given that extracellular ATP strongly inhibits polar auxin transport (Tang et al., 2003; Liu et al., 2012), ATP released by wounding could contribute to the auxin distribution at the wound site. Jasmonic acid and ethylene has been postulated to activate specific wound responsive genes that further promote wound healing (Asahina et al., 2011). The addition of ATP induces the expression of genes involved in the biosynthesis of jasmonic acid and ethylene (Choi et al., 2014). Although definitive experimental evidence is lacking, the literature certainly contains clues that extracellular ATP could be playing a very important signaling role during the wound healing process.

### PLANT–SYMBIONT INTERACTION

The role of extracellular ATP in symbiosis has been suggested by studies of ectoapyrases, which control the level of extracellular nucleotides. For example, overexpression of soybean ectoapyrase in *Lotus japonicus, Medicago truncatula,* and soybean resulted in enhanced infection by rhizobia and an increased number of nodules (McAlvin and Stacey, 2005; Tanaka et al., 2011a). In contrast, RNAi silencing and antisense suppression of ectoapyrases in soybean and *L. japonicus* blocked symbiont infection, resulted in markedly reduced nodulation (Govindarajulu et al., 2009; Roberts et al., 2013). These results are corroborated by earlier studies in which nodule formation on soybean and *Dolichos biflorus* was inhibited by treatment with specific antibodies against ectoapyrases (Etzler et al., 1999; Day et al., 2000; Kalsi and Etzler, 2000). A recent study by Roberts et al. (2013) also demonstrated that the suppression of ectoapyrase blocked infection by arbuscular mycorrhizal fungi. Taken together, these data suggest that ectoapyrases are essential for both rhizobial and mycorrhizal symbiosis, presumably due to their ability to modulate extracellular ATP levels.

Lipochitooligosaccharides (LCOs), which are known as Nod factors and Myc factors, are key symbiotic signals essential for rhizobial and mycorrhizal symbioses, respectively. Treatment of soybean seedlings with LCO was shown to increase ectoapyrase gene expression (Day et al., 2000). LCO treatment was also shown to result in a re-localization of the apyrase to the root hair tip, which is the site of rhizobial infection (Kalsi and Etzler, 2000). Indeed, LCO treatment was shown to increase the enzymatic activity of the ectoapyrase isolated from *D. biflorus* demonstrating that this ectoapyrase has the ability to bind directly to LCO (Etzler et al., 1999). Therefore, one can hypothesize that, in order to enhance infection, the symbionts use LCO to increase ectoapyrase expression and/or activity; thereby quenching DAMP, extracellular ATP, either released due to mechanical stress during infection (Jayaraman et al., 2014) or as a result of a response to LCO (Tanaka et al., 2011a,b). Studies have shown that ATP, but not ADP, induces ROS production in the root hairs of *Medicago truncatula* (Kim et al., 2006) and *Arabidopsis* (Clark et al., 2010b), as well as *Arabidopsis* epidermal cells (Demidchik et al., 2011). ROS are well-known signals in the plant defense response. Therefore, symbionts may control ectoapyrase activity using LCOs, in which the ATP function as a DAMP is suppressed so that plant defenses are not triggered.

The products of ectoapyrase activity are ADP and/or AMP, which may also have biological activity. For example, ADP was shown to change plasma membrane conductance in root hairs (Lew and Dearnaley, 2000) and induce calcium influx in root epidermal cells (Demidchik et al., 2011). Interestingly, while RNAi silencing of ectoapyrase expression in soybean reduced nodulation, this effect was partially reversed by the addition of exogenous ADP, suggesting a role for extracellular ADP in the nodulation process. Demidchik et al. (2011) used electrophysiological methods to verify the presence of an ADP signaling pathway, independent of ATP signaling, with ionotrophic receptor-like activity at the plasma membrane. P2K1 (DORN1) shows slightly less affinity for ADP over ATP (Choi et al., 2014). It is possible that a second, distinct receptor could exist in plants for ADP, as suggested by Demidchik et al. (2011). Alternatively, it is also possible that ADP recognition by P2K1 could activate distinct downstream signaling events, which would also account for the apparent distinct response of ADP vs. ATP. Further research on ADP/ATP signaling pathways, possible additional purinoceptors and P2K1 function should provide answers to these and other questions relevant to nucleotide signaling in plants.

## FUTURE PERSPECTIVES OF EXTRACELLULAR ATP AS A DAMP SIGNAL

The release and response to extracellular ATP is conserved across many taxa, including lower vertebrates, insects, protozoa, and prokaryotes (Burnstock, 1996; Burnstock and Verkhratsky, 2009), indicating that a role for ATP as an extracellular signal is an ancient trait. Therefore, it is not surprising that ATP also plays such a role in plants. However, the P2K purinoceptor family is quite distinct from the P2X and P2Y receptors found in animal systems. Hence, although ATP signaling is widely conserved, the means by which plants and animals recognize this signal appears to have evolved independently. However, caution should be used in making such broad conclusions since we are clearly at a very early stage of our understanding of ATP recognition and action in plants. Additional comparative studies are needed to fully elucidate the similarities and differences between the mechanism of action of extracellular ATP in plants and animals.

Plant enemies such as pathogens and insects usually interfere with one or more defense signals to promote colonization and infection. It is now clear that among these mechanisms are strategies for modulating the levels of extracellular ATP. This underlines the important role that ATP plays in general plant stress response (**Figure 1**).

Although a number of molecular components need to be identified for a complete understanding of extracellular ATP signaling, the recent discovery of the P2K1 (DORN1) purinoceptor (Choi et al., 2014) creates the possibility for further genetic and biochemical studies. As shown in **Figure 2**, the repertoire of the molecular components identified to date covers a variety of responses induced by extracellular ATP. The following research avenues are of particular interest for further investigation: (1) understanding how these molecular components are controlling extracellular ATP levels following various environmental stimulations and (2) learning the molecular mechanisms of how the ATP signal influences the immune system and subsequent wound healing processes. We propose that extracellular ATP is a key missing signal in many plant stress responses that were previously believed to be due to a direct effect of the stress stimulus itself. Therefore, further knowledge of the role of ATP in plants is crucial to understanding how plants adapt to environmental changes, a crucial question in lieu of a rapidly changing climate.

## Conflict of Interest Statement

The authors declare that the research was conducted in the absence of any commercial or financial relationships that could be construed as a potential conflict of interest.
